# Evaluation of metagenetic community analysis of planktonic copepods using Illumina MiSeq: Comparisons with morphological classification and metagenetic analysis using Roche 454

**DOI:** 10.1371/journal.pone.0181452

**Published:** 2017-07-17

**Authors:** Junya Hirai, Satoshi Nagai, Kiyotaka Hidaka

**Affiliations:** National Research Institute of Fisheries Science, Japan Fisheries Research and Education Agency, Yokohama, Kanagawa, Japan; University of Shiga Prefecture, JAPAN

## Abstract

Metagenetics is a rapid and taxonomically comprehensive method for revealing community structures within environmental samples, based on large amounts of sequence data produced by high-throughput sequencers. Because community structures of planktonic copepods are important in the ocean owing to their high diversity and abundance, a metagenetic analysis of the 28S D2 region using Roche 454 was previously developed. However, the Illumina MiSeq platform with a high sequence output is being used more frequently in metagenetics, and non-calanoid copepods have not previously been fully evaluated. Here, we evaluated an Illumina MiSeq-based metagenetic analysis using a mock community and field-collected samples that were examined in a previous study using Roche 454, and the community structure, including non-calanoid copepods, was compared among morphological and metagenetic analyses. We removed a singleton read and applied an appropriate abundance threshold to remove erroneous Molecular Operational Taxonomic Units (MOTUs) with low-abundance sequences in the MiSeq-based analysis. Results showed that the copepod community was successfully characterized using Illumina MiSeq. Higher-quality sequences were obtained using MiSeq than by Roche 454, which reduced the overestimation of diversity, especially at a strict 99% similarity threshold for MOTU clustering. Taxonomic compositions in terms of both biomass and presence/absence of species, including non-calanoids, were more appropriately represented in the MiSeq- than in Roche 454-based analysis. Our data showed that metagenetic analysis using Illumina MiSeq is more useful for revealing copepod communities than Roche 454, owing to the lower cost and higher quality.

## Introduction

Metagenetics examines community structures within environmental samples, based on the large amount of sequence data produced by high-throughput sequencers. This method has been primarily developed in microorganisms using a target gene, such as 16S or 18S ribosomal RNA (rRNA) [1–3], and applied to metazoan communities, such as benthic meiofauna [4], terrestrial arthropods [5], and fish communities [6]. Although morphological classification is commonly used to investigate the community structures of zooplankton, which play a significant role in marine ecosystems, DNA-based approaches accurately identify specimens, including immature stages and cryptic species [7]. Morphological classification and DNA-based identification using Sanger sequencing are timeconsuming; however, metagenetic analysis is a rapid and taxonomically comprehensive method to reveal zooplankton communities in the ocean [8–11].

Of the high-throughput sequencers, the Roche 454 platform was the first to be introduced and used as a method to detect the hidden diversity of microorganisms [1, 12]. Compared with other platforms, Roche 454 provided relatively long sequences, suitable for amplicon sequencing of specific target regions. In contrast, Illumina MiSeq with a high sequence output, which is becoming more common in metagenetics, produces relatively short sequences that limits their use for metagenetic analysis. However, 2 × 300 bp paired-end sequencing is now available from Illumina MiSeq, which can be adapted to various target-gene metagenetic analyses. Because Illumina MiSeq can produce more than 10 times the number of sequence reads of Roche 454 at a lower cost, metagenetic analysis using Illumina MiSeq is a relatively cost-effective method that is being used more frequently [13, 14]. Data types are different for each platform, and there are platform-specific sequencing errors, such as frequent homopolymer errors from Roche 454 and substitution errors from Illumina MiSeq [15, 16]. Comparisons between the different platforms have been conducted using 16S [17] and 18S rRNA genes [18] to examine microbial communities, as well as metagenomic studies of microbial communities [19]. These studies represent comparable overviews between Illumina and Roche 454 systems using samples of the same community, although there are some platform-specific analytical results owing to different sequencing outputs and bioinformatics procedures. As Illumina-based community analysis becomes more common, it will be important to confirm if metagenetic methods previously developed for Roche 454 can be comparable with Illumina-based analyses.

Marine planktonic copepods are one of the most diverse and abundant zooplankton groups, an informative indicator of environmental changes, and a major prey for higher tropic levels, such as fish larvae [20]. The Roche 454-based metagenetic method was previously used with the 28S D2 region [21]; however, application of the metagenetic method using Illumina MiSeq was not evaluated in previous studies. In addition, metagenetic data were only compared with morphological classifications of the order Calanoida, although other orders (e.g., Poecilostomatoida or Cyclopoida) also play a significant role in the marine ecosystem, especially in communities of small (< 1.0 mm) plankton [22, 23]. Therefore, evaluating MiSeq-based metagenetic analysis across a wider diversity of copepods would aid future community analysis of copepod communities.

In this study, we performed Illumina MiSeq-based metagenetic analysis of copepods using samples previously used for Roche 454-based metagenetic analysis. Taxonomic compositions of non-calanoid copepods based on morphological analysis were additionally collected, and metagenetic analyses of the 28S D2 region using data from both platforms were compared with a morphological analysis of copepods. Based on the results of these comparisons, the metagenetic approach using Illumina MiSeq was evaluated as a method for accurately revealing copepod diversity within planktonic communities.

## Materials and methods

### Sample information

Morphologically identified mock community and field-collected samples from the Kuroshio region from a previous study [21] were used for the MiSeq analysis in this study. The mock community was an artificially prepared community sample containing 33 morphologically identified copepod species, including 30 Calanoida, 1 Cyclopoida, and 2 Poecilostomatoida. One individual of each species included in the mock community sample was used to evaluate bioinformatics procedures. Zooplankton samples were collected in the field at 0–200 m depth using a VMPS net with a 0.25 m^2^ mouth-opening area and 100 μm mesh at three stations (Slope, Kuroshio, and Subtropical) along the monitoring line across the Kuroshio Current at 138°E off the southern coast of Japan [24]. The samples were not collected in a protected area. No specific permissions were required to conduct the sampling, as this study did not involve endangered or protected species. The samples were split, and aliquots were used to compare the morphological classification and metagenetic analysis. Although the previous study used only Calanoida for morphological classification of field-collected samples, morphological data for non-calanoid copepods, including Cyclopoida, Harpacticoida, and Poecilostomatoida, were included in this study. Wet weights of non-calanoid species were calculated and converted into dry weights according to Shmeleva [25] and Harris et al. [26].

### High-throughput sequencing

Metagenetic data using the Illumina MiSeq platform were obtained using DNA extracted in the previous study [21]. Three stages of PCR were used to amplify the 28S D2 region (approximately 400 bp) and yield libraries for Illumina MiSeq, using the primer pairs listed in Table 1. First, PCR using the KOD Plus Version 2 high-fidelity polymerase (Toyobo) was prepared in a 25 μL volume that contained 13 μL distilled water, 2.5 μL 10× buffer, 2.5 μL dNTPs (2 mM), 1.5 μL MgSO_4_ (25 mM), 1.5 μL of each primer (5 μM), 0.5 μL KOD Plus polymerase, and 2 μL template DNA (1 ng/μL). PCR cycling included initial denaturation at 94°C for 1 min, followed by 22 cycles of 10 s denaturation at 98°C, 15 s annealing at 58°C, and 1 min extension at 68°C. A final extension step was performed at 68°C for 7 min. The PCR product was diluted (1:20) for the second and third PCRs, which attached MiSeq adaptors and dual-index target sequences to discriminate samples. The PCR programs used eight cycles, with an annealing temperature of 50°C for the second PCR and 59°C for the third PCR. Final PCR products were purified using the QIAquick PCR Purification Kit (QIAGEN), and the concentration of purified PCR product was measured using a Qubit 3.0 Fluorometer (Life Technologies). The final PCR products were transferred to FASMAC Co. Ltd., to perform a single sequencing run using MiSeq Reagent Kit v3 on Illumina MiSeq to obtain 2 × 300 bp paired-end sequences (accession number in the NCBI/EBI/DDBJ Sequence Read Archive: DRA004811).

**Table 1:** PCR primers used in this study.

Primer name	Platforms	Sequence
LSU Cop-D2F [21]	MiSeq & Roche 454	5´-AGACCGATAGCAAACAAGTAC-3´
LSU Cop-D2R [21]		5´-GTCCGTGTTTCAAGACGG-3´
2nd primer for LSU Cop-D2F	MiSeq	5´-ACACTCTTTCCCTACACGACGCTCTTCCGATCTAGACCGATAGCAAACAAGTAC-3´
2nd primer for LSU Cop-D2R		5´-GTGACTGGAGTTCAGACGTGTGCTCTTCCGATCTGTCCGTGTTTCAAGACGG-3´
3rd forward primer	MiSeq	5´-AATGATACGGCGACCACCGAGATCTACACXXXXXXXXACACTCTTTCCCTACACGACGC-3´
3rd reverse primer		5′-CAAGCAGAAGACGGCATACGAGATXXXXXXXXGTGACTGGAGTTCAGACGTGTG-3′

Sequencing adaptors and index tags to discriminate samples were attached during second and third PCRs for metagenetic analysis using Illumina MiSeq. The third round of PCR primers contained an 8-bp index (represented as XXXXXXXX) to discriminate between samples. We used an index of AGGCGAAG in the 3rd forward primer and GCGCATTA in the 3rd reverse primer for the mock community. The same index of TTCGCGGA was used in the 3rd reverse primer for three field-collected samples, and a different index was used in the 3rd forward primer: CCTATCCT (Slope), TAATCTTA (Kuroshio), and TAGATCGC (Subtropical).

We also used previous sequence data acquired using Roche 454 (Accession number in the NCBI/EBI/DDBJ Sequence Read Archive: DRA002161) from Hirai et al. [21]. Adaptor and index tags were attached by ligation for Roche 454 after the first PCR using Tks Gflex DNA Polymerase (Takara) in the previous study, which were the main differences from samples prepared for the MiSeq-based analysis in this study.

### Bioinformatics procedures

Sequence data from Illumina MiSeq and Roche 454 were analyzed separately, with some modifications from the previous studies [21, 27]. A total of four samples (one mock community and three field-collected samples) were analyzed simultaneously in each bioinformatics analysis of MiSeq and Roche 454 data. Raw paired-end reads from Illumina MiSeq were initially quality-filtered using Trimmomatic [28] based on the following settings: CROP:300 MINLEN:100 LEADING:20 TRAILING:20 SLIDINGWINDOW:30:30. Quality-filtered paired-end reads were merged and used for the subsequent bioinformatics analysis in MOTHUR [29]. Merged sequence reads were again quality-filtered based on the following criteria: (i) contained no ambiguous bases (Ns); (ii) comprised 300–420 bp not including the primer sites; (iii) contained ≤ 5 homopolymers; (iv) contained no more than three mismatches per primer; and (v) contained primer sites. Primer sites were removed using the pcr.seqs command for the sequence data in Illumina MiSeq-based analysis. We classified sequence reads into taxonomic groups using a naïve Bayesian classifier [30] with a threshold > 70%. Only copepod sequences subsampled based on minimum sequence reads, with the add-fragments option in Multiple Alignment using Fast Fourier Transform (MAFFT), were used for sequence alignments with the default setting [31]. The reference sequences for taxonomic classifications and alignment were updated in this study (S1–S3 Files). Singleton reads were removed after single-linkage pre-clustering in MiSeq data [32] as suggested by Unno [33]. Possible chimeras were removed by UCHIME both with and without a reference dataset [34]. We used a template file for taxonomic classification as a reference dataset in UCHIME. To remove contaminants of non-copepod sequences, the taxonomic classification of all sequences was checked again. The final quality-filtered sequences were subsampled and clustered into Molecular Operational Taxonomic Units (MOTUs) at the 97 and 99% similarity thresholds using the average neighbor algorithm in MOTHUR. Insertions and deletions were removed from distance calculations. The representative sequence of each copepod MOTU was obtained based on the most abundant sequence reads.

The data from Roche 454 were analyzed in this study to compare with data from Illumina MiSeq. As the same quality-filtering criteria of ≥ 30 within any 30-bp sequences led to reductions of most sequence reads, an average quality score of > 27 was used for metagenetic data from Roche 454, according to Hirai et al. [21]. Contrary to the MiSeq analysis, singleton reads were not removed after pre-clustering, because this step was not used in the previous Roche 454-based study [21] and led to significant reductions of sequence data including target species. Primer sites were removed using the screen.seqs command after alignment of sequence data. Other quality filtering criteria and methods for clustering of MOTUs were the same as those in the analysis of MiSeq data.

### Data analysis of mock community and field-collected samples

An appropriate abundance threshold for MOTUs was evaluated based on results of the mock community analyses, to avoid overestimations of diversity by erroneous MOTUs in both MiSeq and Roche 454 analyses. After applying the abundance threshold, MOTU numbers were compared with reference sequences of 33 morphological species. The clustered MOTUs were treated as ‘selected MOTUs’ derived from 33 morphological species, if the representative sequences showed higher similarity to at least one of the 33 reference sequences at a specific threshold (97 or 99%) and as ‘non-selected MOTUs’ if they did not.

In the metagenetic analyses of field-collected samples, the abundance threshold determined in the mock analysis was applied, and MOTUs at 97 and 99% similarity thresholds were compared with species numbers in the morphological analysis. Order- and family-level taxonomic compositions of MOTUs were also evaluated at a 97% similarity threshold, because this similarity threshold was used in the previous Roche 454-based study [21]. As dry weight was correlated with sequence reads [21], proportions of sequence reads were compared with those of dry weight from morphological data in taxonomic orders and families. Pearson’s product moment correlation coefficients (*r*) were calculated for numbers of morphological species and MOTUs, as well as for proportions of dry weight and sequence reads. Statistical analyses were performed using SPSS version 21.0 (IBM Corporation).

The dominant species of field-collected samples were also compared between morphological and metagenetic analyses. The representative sequences of MOTUs at 99% similarity threshold were blasted against the NCBI database and were assigned to a specific species if they had ≥ 99% similarity to registered sequences of copepods. The top 12 dominant species based on biomass or sequence read proportions were selected. In the morphological analysis, proportions of numbers of individuals were also used for comparison.

## Results

### Mock community analysis

**Fig 1:**
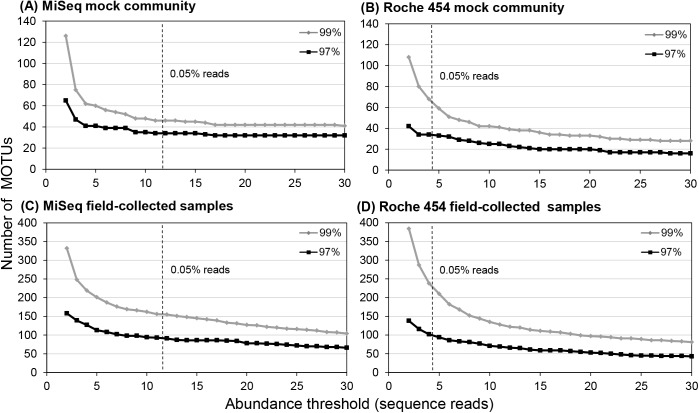
MOTU numbers at different minimum abundance thresholds of sequence reads: Comparisons of mock community analysis using (A) Illumina MiSeq and (B) Roche 454, and analyses of field-collected samples using (C) Illumina MiSeq and (D) Roche 454. A 0.05% of total reads for a single sample is indicated by dashed lines. Effects of the abundance threshold on MOTU numbers were evaluated both at a 97 and 99% similarity threshold for MOTU clustering.

After quality-filtering and subsampling of sequence reads before MOTU clustering, a total of 23,556 reads were obtained from Illumina MiSeq and 8,685 reads from Roche 454 in each sample. In the mock community analyses, we observed surplus MOTUs, mainly caused by rare MOTUs with small sequence reads derived from dominant species, leading to larger numbers of MOTUs than expected at a small number of abundance thresholds for both MiSeq and Roche 454 analyses. MOTU numbers decreased with increasing numbers of abundance thresholds (Fig 1). The total MOTU number including singletons was 67 (188 if singleton reads were not removed) using MiSeq and 80 MOTUs using Roche 454 before applying the abundance threshold at a 97% similarity threshold. MOTU numbers with at least two sequence reads were 65 (MiSeq) and 42 (Roche 454) at a 97% similarity threshold. Minimum sequence reads of MOTUs derived from selected species were 15 for Illumina MiSeq (*Metridia brevicauda*) and 4 for Roche 454 (*Calocalanus* sp.) at a 97% similarity threshold. Therefore, the minimum abundance threshold for MOTUs was set to four reads for Roche 454 (0.046% sequence reads) in this study. At this abundance threshold of four sequence reads, we still observed surplus MOTUs in the MiSeq analysis (41 MOTUs at 97% similarity threshold). For comparisons between two different sequence platforms, similar criteria of a minimum of 11 sequence reads (0.047% sequence reads) were applied as the abundance threshold for the following Illumina MiSeq-based analyses.

After applying the determined abundance thresholds, we obtained 34 MOTUs at a 97% similarity threshold using MiSeq and Roche 454 (Table 2; S1 Fig). Several genetically related species were clustered into the same MOTU, and 27 MOTUs were expected as numbers of selected MOTUs based on Sanger sequencing at 97% similarity threshold. *Metridia venusta* were undetected in both MiSeq and Roche 454 analyses, and *Oncaea* sp. was detected only in the MiSeq analysis. We detected 26 and 27 MOTUs in MiSeq and Roche 454 analyses, respectively. Detected MOTUs in the MiSeq analysis were almost the same as expected MOTUs, whereas Roche 454 analysis showed surplus MOTUs in Calanidae and Eucalanidae. There were 8 and 7 non-selected MOTUs in MiSeq and 454 analyses, respectively, and 6 MOTUs were shared (S1 Fig). Percentages of sequence reads at a 97% similarity threshold were significantly correlated between MiSeq and Roche 454 (*r* = 0.939, *P* < 0.01). MOTUs derived from dominant species with a large biomass tended to represent high proportions of sequence reads (S1 Fig), leading to significant correlations between biomass in the morphological analysis and sequence reads in metagenetic analyses (MiSeq: *r* = 0.548, *P* < 0.01; Roche 454: *r* = 0.674, *P* < 0.01).

**Table 2:** Lists of species and MOTUs in the mock community analysis.

		97% similarity			99% similarity		
Taxon	Species	Sanger	MiSeq	454	Sanger	MiSeq	454
Order Calanoida							
Aetideidae	*Aetideus acutus*	○	○	○	○	○	○(2)
	*Euchirella curticauda*	○	○	○	○	○	○(2)
	*Euchirella messinensis*	○	○	○	○	○	○(5)
	*Gaetanus minor*	○	○	○	○	○	○
	*Undeuchaeta major*	○	○	○	○	○(3)	○(3)
	*Undeuchaeta plumosa*						
Augaptilidae	*Haloptilus* sp.	○	○	○	○	○	○(2)
Calanidae	*Calanus sinicus*	○	○	○(2)	○	○	○(2)
	*Cosmocalanus darwini*				○	○	○(2)
	*Mesocalanus tenuicornis*				○	○	○
	*Nannocalanus minor*				○	○	○(2)
	*Neocalanus gracilis*	○	○	○	○	○	○
Candaciidae	*Candacia curta*	○	○	○	○	○	○(2)
Centropagidae	*Centropages* sp.	○	○	○	○	○	○(2)
Eucalanidae	*Eucalanus californicus*	○	○	○	○	○	○(3)
	*Pareucalanus* sp.	○	○	○(2)	○	○	○(2)
	*Pareucalanus attenuatus*				○	○	○(2)
	*Subeucalanus subtenuis*	○	○	○	○	○	○(3)
Euchaetidae	*Paraeuchaeta media*	○	○	○	○	○	○(2)
Lucicutiidae	*Lucicutia flavicornis*	○	○	○	○	○	○
Mecynoceridae	*Mecynocera clause*	○	○	○	○	○	○
Metridinidae	*Metridia brevicauda*	○	○	○	○	○	○
	*Metridia venusta*	○	×	×	○	×	×
	*Pleuromamma abdominalis*	○	○	○	○	○(2)	○(2)
	*Pleuromamma gracilis*				○	○	×
Paracalanidae	*Calocalanus* sp.	○	○	○	○	○	○
	*Paracalanus* sp.	○	○	○	○	○	○
Pontellidae	*Pontellina plumata*	○	○	○	○	○	○(2)
Scolecitrichidae	*Scolecithrix danae*	○	○	○	○	○	○
Temoridae	*Temora discaudata*	○	○	○	○	○	○(3)
Order Cyclopoida							
Oithonidae	*Oithona* sp.	○	○	○	○	○	○
Order Poecilostomatoida							
Corycaeidae	*Corycatus* sp.	○	○	○	○	○	○
Oncaeidae	*Oncaea* sp.	○	○	×	○	○	×
Selected MOTUs		/	26	27	/	34	53
Non-selected MOTUs		/	8	7	/	12	15
Total MOTUs		27	34	34	32	46	68

Reference sequences of 33 copepod species by Sanger sequencing were compared with MOTUs at 97 and 99% similarity thresholds in metagenetic analyses using Illumina MiSeq and Roche 454. Results for species that are expected to be clustered into a single MOTU are surrounded by a box. Both detected (○) and undetected (×) species are shown in each metagenetic analysis. Where ≥ 2 MOTUs were identified, numbers of MOTUs are indicated within parentheses in each expected MOTU. Non-selected MOTUs are not identified in any of the 33 morphological species.

At a 99% similarity threshold, there was higher species-level resolution than at a 97% similarity, as shown in expected MOTUs by reference sequences (Table 2). A total of 46 and 68 MOTUs were obtained at a 99% similarity threshold for MiSeq and Roche 454 analyses, respectively, and higher taxonomic resolution was observed compared with a 97% similarity threshold (Table 2). Overestimation of MOTUs was only found in two expected MOTUs in the MiSeq analysis, and other species showed > 99% similarity to a single MOTU in the MiSeq analysis. Overestimations of diversity were especially evident in the Roche 454 analysis, and most species showed > 99% similarity to several MOTUs.

### Diversity analysis of field-collected samples at a 97% similarity threshold

**Fig 2:**
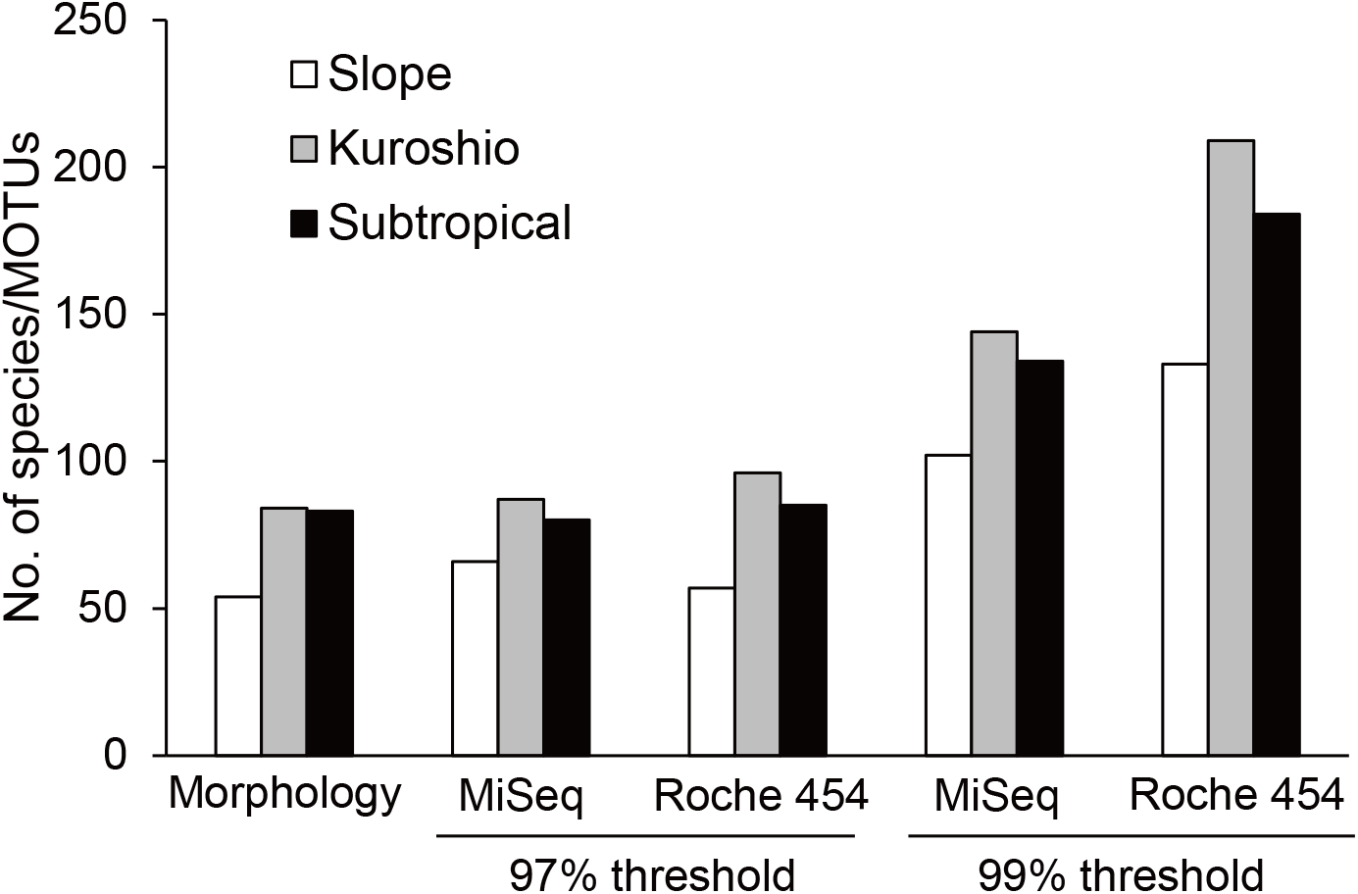
Total species and MOTU numbers of copepods in field-collected samples. MOTU numbers were calculated at 97 and 99% similarity thresholds in metagenetic analyses using both Illumina MiSeq and Roche 454 data.

The total number of morphological species at each station was 54 (Slope), 84 (Kuroshio), and 83 (Subtropical) (Fig 2). After applying the same abundance threshold as in the mock community analysis, the same diversity patterns were observed in both MiSeq and Roche 454 analyses, with the highest number of MOTUs at the Kuroshio station, followed by the Subtropical and Slope stations. MOTU numbers at each station were similar in both MiSeq and Roche 454 analyses at a 97% similarity threshold: 66 (Slope), 87 (Kuroshio), and 80 (Subtropical) MOTUs for MiSeq, and 57 (Slope), 96 (Kuroshio), and 85 (Subtropical) for Roche 454. The total species number was 122 in the morphological analysis, which was higher than the total MOTU numbers of 93 and 102 in MiSeq and Roche 454 analyses, respectively (Table 3).

**Table 3:** Comparison of morphologically classified species and MOTU numbers at 97 and 99% similarity thresholds in field-collected samples.

			97% similarity		99% similarity	
Order	Family	Morphology	MiSeq	454	MiSeq	454
Calanoida	Acartiidae	3	0	1	0	1
	Aetideidae	2	2	2	2	3
	Augaptilidae	2	1	4	1	2
	Calanidae	6	3	4	9	21
	Candaciidae	2	5	5	7	9
	Centropagidae	0	4	3	5	4
	Clausocalanidae	8	2	3	5	15
	Eucalanidae	8	7	7	10	28
	Euchaetidae	4	2	4	7	10
	Fosshageniidae	1	1	1	1	2
	Heterorhabdidae	1	1	1	1	1
	Lucicutiidae	3	3	4	5	10
	Mecynocenidae	1	1	1	1	1
	Metridinidae	2	2	2	4	6
	Paracalanidae	19	14	15	29	31
	Pontellidae	0	1	1	1	1
	Rhincalanidae	2	2	2	2	4
	Scolecitrichidae	6	5	4	6	4
	Spinocalanidae	1	0	0	0	0
	Temoridae	2	2	2	2	3
	Unclassified	0	1	1	1	3
Cyclopoida	Oithonidae	13	9	10	18	44
Harpacticoida	All families	4	4	3	5	4
Poecilostomatoida	Corycaeidae	11	5	5	7	8
	Lubbockiidae	0	1	1	1	1
	Oncaeidae	20	9	7	15	13
	Sapphirinidae	1	1	1	1	1
	Unclassified	0	3	3	6	3
Unclassified		0	2	5	4	5
Total		122	93	102	156	238

Species richness in taxonomic families was compared in each taxonomic order, except Harpacticoida.

**Fig 3:**
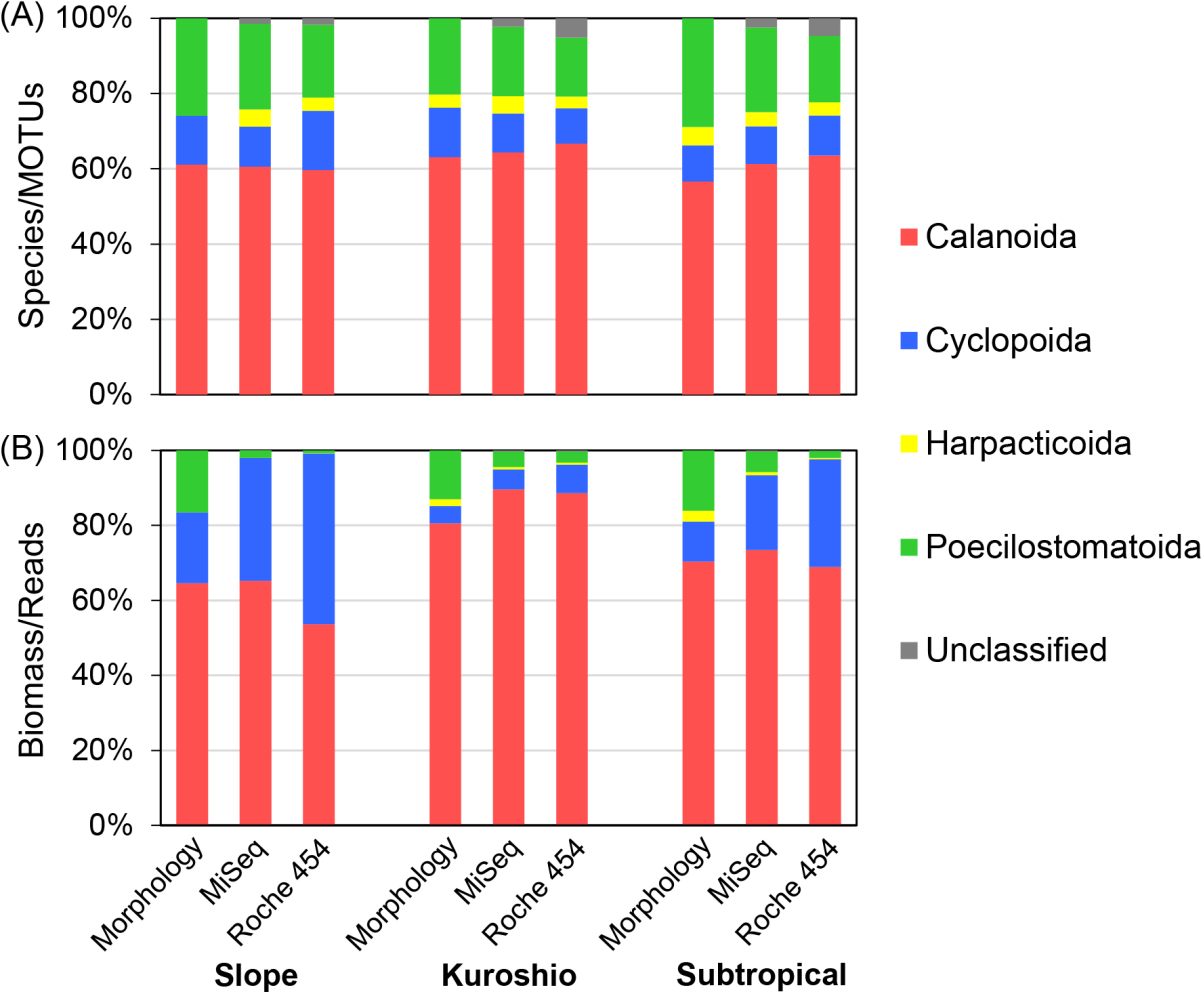
**Order-level taxonomic compositions of copepods in field-collected samples between (A) species and MOTUs, and (B) biomass and sequence reads.** Metagenetic analyses using both Illumina MiSeq and Roche 454 data were performed at a 97% similarity threshold.

Order-level taxonomic compositions from the morphological analysis were clearly reflected in MiSeq and Roche 454 analyses (Fig 3A). The highest diversity was in calanoid copepods, followed by Poecilostomatoida, Cyclopoida, and Harpacticoida. These order-level MOTUs and species numbers were significantly correlated between all analyses for each station (Table 4). The diversity of the order Poecilostomatoida was higher in the MiSeq than Roche 454 analysis at all stations (Fig 3A), which more appropriately represented taxonomic compositions by morphological analysis.

**Table 4:** Pearson correlation coefficients between different methods of morphological and metagenetic analyses.

		Correlation (species or MOTU numbers/biomass or sequence reads)		
Taxon	Station	Morphology-MiSeq	Morphology-454	MiSeq-454
Order	Slope	0.992**/0.937*	0.986*/0.812	0.991**/0.954*
	Kuroshio	0.998**/0.993**	0.996**/0.990**	0.996**/0.999**
	Subtropical	0.987*/0.972**	0.965*/0.923*	0.993**/0.986**
Family	Slope	0.446/0.888**	0.501/0.912**	0.877**/0.993**
(Calanoida)	Kuroshio	0.906**/0.716**	0.889**/0.825**	0.953**/0.980**
	Subtropical	0.854**/0.796**	0.874**/0.920**	0.942**/0.922**

**Fig 4:**
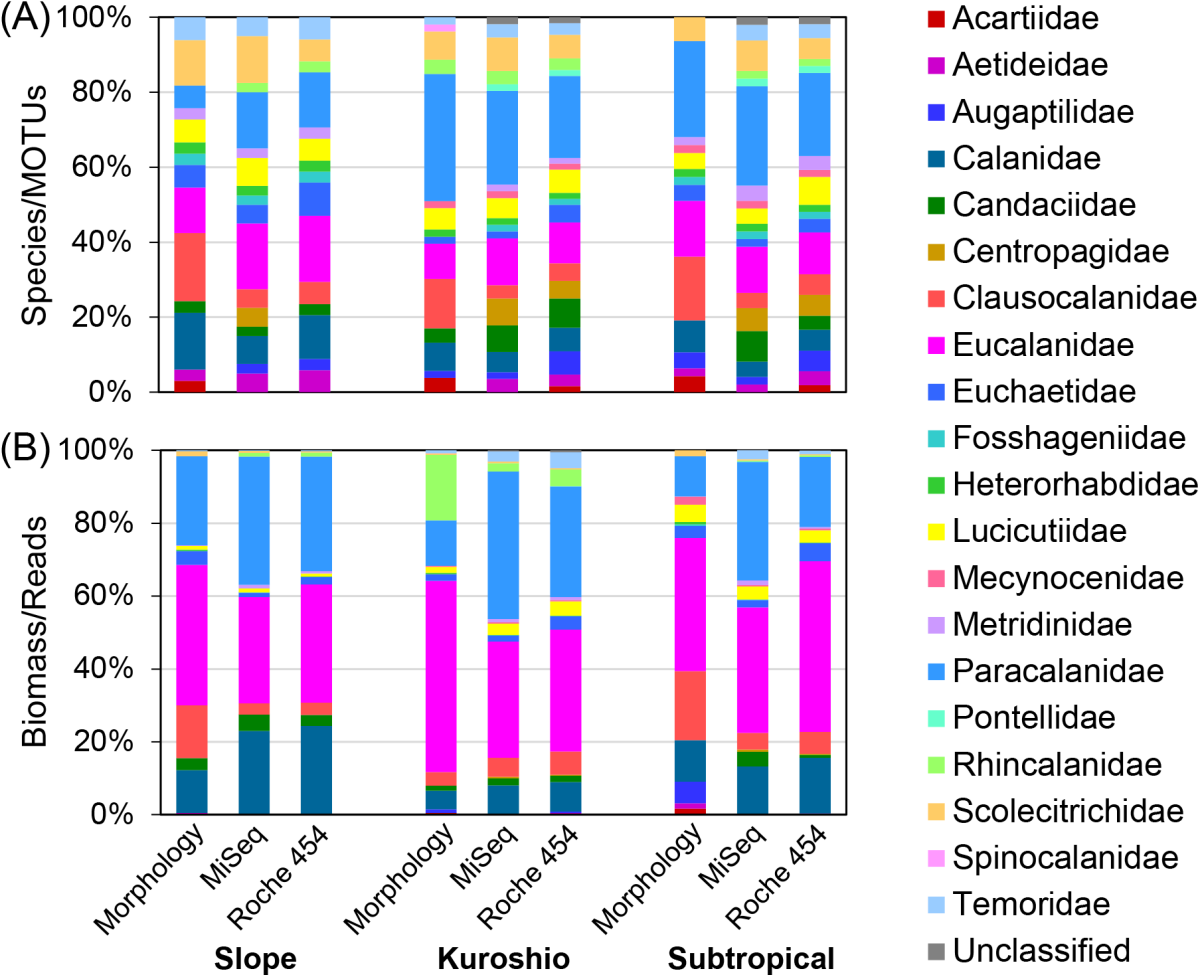
**Family-level taxonomic compositions of calanoid copepods in field-collected samples between (A) species and MOTUs, and (B) biomass and sequence reads.** Metagenetic analyses using both Illumina MiSeq and Roche 454 data were performed at a 97% similarity threshold.

Both species/MOTU number (left) and biomass/sequence reads (right) were correlated at each station. Comparisons were performed at both the order-level and family-level of taxonomic groups, which are shown in Figs 3 and 4. Significant correlations are indicated by asterisks (*P < 0.05; ** *P* < 0.01).

Family-level taxonomic compositions within Calanoida were also highly similar between morphological and metagenetic analyses, with significant correlations at all stations (Fig 4A; Table 4). Total MOTU numbers in the taxonomic families within Calanoida were almost the same between MiSeq and Roche 454 analyses (Table 3). MiSeq and Roche 454 analyses showed significant correlations with family-level taxonomic compositions from morphological analysis at Kuroshio and Subtropical stations (Table 4). The total species number was underestimated in some families at a 97% similarity threshold in metagenetic analyses, including Acartiidae, Calanidae, Clausocalanidae, and Paracalanidae (Table 3). The family Acartiidae (3 morphological species) was not detected in the MiSeq analysis, and only one MOTU was observed in the Roche 454 analysis. The families Centropagidae and Pontellidae were only found in the metagenetic analyses. In non-calanoid copepods, the order Harpacticoida was not analyzed at the family level owing to the lack of a reference database. In the order Poecilostomatoida, diversity patterns were the same in morphological and metagenetic analyses, with the highest diversity in Oncaeidae, followed by Corycaeidae. Three unclassified MOTUs of Poecilostomatoida were observed both in MiSeq and Roche 454 analyses.

### Diversity analysis of field-collected samples at a 99% similarity threshold

A 99% similarity threshold led to higher MOTU numbers than morphologically classified species at each station: 102 (Slope), 144 (Kuroshio), and 134 (subtropical) MOTUs for MiSeq, and 133 (Slope), 209 (Kuroshio), and 184 (subtropical) for Roche 454 (Fig 2). Total MOTU numbers at a 99% similarity threshold were 156 in MiSeq and 238 in Roche 454 analysis (Table 3). The increases in MOTU numbers were especially evident for taxonomic families, in which numbers of species were underestimated at a 97% similarity threshold. When the similarity threshold was changed from 97 to 99% in the MiSeq-based analysis, total MOTU numbers increased from 3 to 9 in Calanidae (6 morphological species), 2 to 5 in Clausocalanidae (8 morphological species), 14 to 29 in Paracalanidae (19 morphological species), 9 to 18 in Oithonidae (13 morphological species), and 9 to 15 in Oncaeidae (20 morphological species). In the Roche 454-based analysis at a 99% similarity threshold, surplus of MOTUs to number of species obtained by morphological analysis was evident especially in dominant taxonomic groups, such as Calanidae (21 MOTUs and 6 species), Eucalanidae (28 MOTUs and 8 species), and Oithonidae (44 MOTUs and 13 species).

### Comparison between biomass and sequence reads of taxonomic groups in field-collected samples

The order-level comparisons between biomass and sequence reads showed the highest percentages of Calanoida both in morphological and metagenetic analyses at all stations (Fig 3B). Percentages of Cyclopoida were higher in the metagenetic (5.3−32.7/7.6−45.5% in MiSeq/Roche 454 analysis) than morphological (4.6−18.8%) analyses. Conversely, percentages of Poecilostomatoida were smaller in the metagenetic (2.0−5.5/0.8−3.0% in MiSeq/Roche 454 analysis) than morphological (13.0−16.6%) analyses. Proportions of Cyclopoida and Poecilostomatoida in biomass by morphological analysis were more similar to sequence reads in the MiSeq-based analysis than to those in the Roche 454-based analysis. Harpacticoida had low percentages of biomass (≤ 2.9%) and sequence reads in all samples (≤ 0.8 and ≤ 0.5% in MiSeq and Roche 454 analysis respectively). The order-level quantitative data were significantly correlated between morphological and metagenetic analyses, except for the Roche 454-based analysis at Slope station (Table 4). There were highly significant correlations in all comparisons between order-level MiSeq and Roche 454 analyses.

Family-level analysis of Calanoida also showed the same dominant taxa between morphological and metagenetic analyses, including Calanidae, Eucalanidae, and Paracalanidae (Fig 4B). There were significant correlations in family-level compositions between morphological and metagenetic analyses, as well as between MiSeq-based and Roche 454-based analyses.

### Comparison of dominant species between morphological and metagenetic analyses

**Fig 5:**
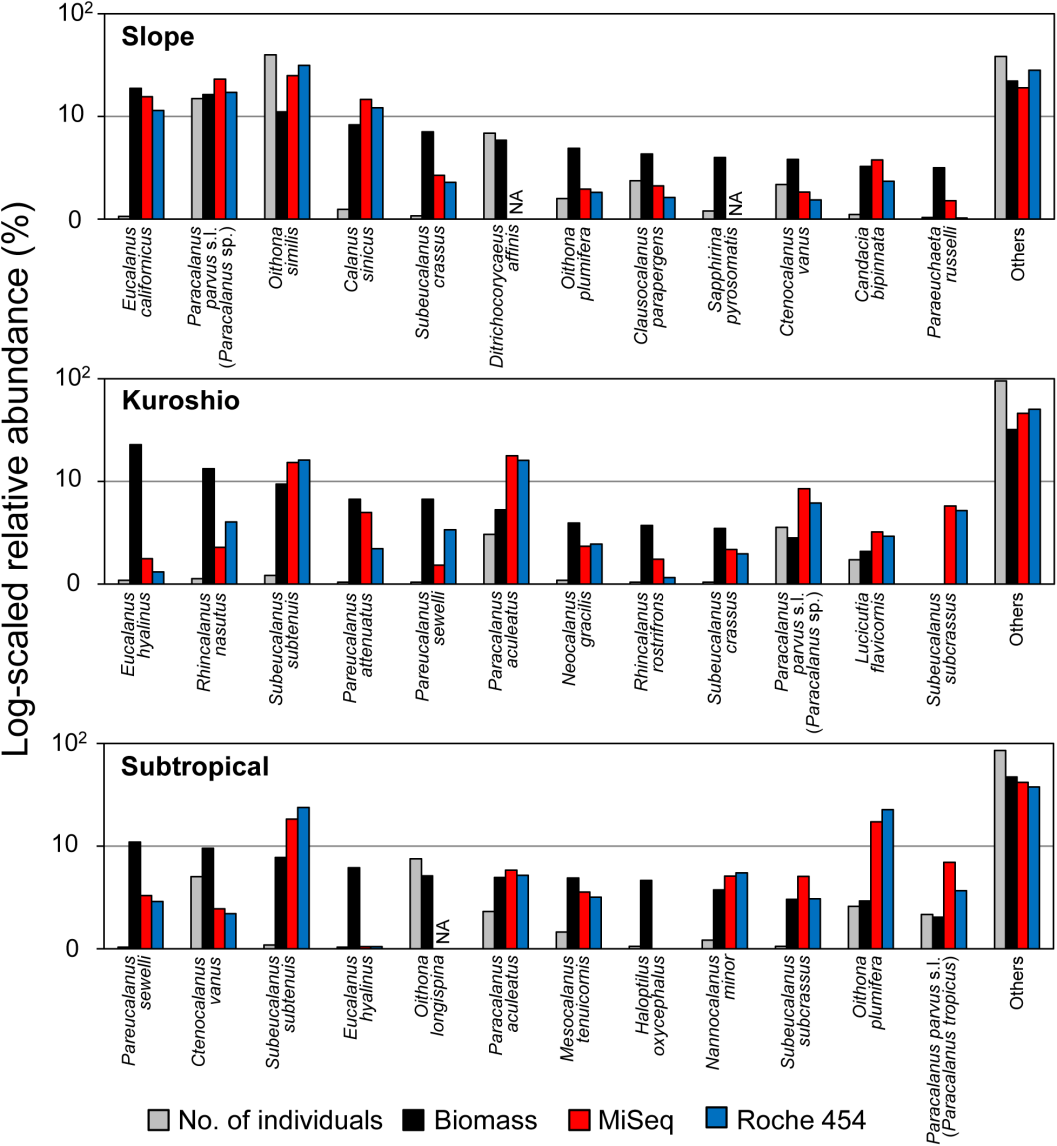
Proportions of dominant species and MOTUs at each station. MOTUs at a 99% similarity threshold were assigned to species. NA represents MOTUs which were not assigned to any copepod species (< 99% similarity to public database). The proportions of numbers of individuals, biomass, and sequence reads are presented as log_x+1_-transformed values. The morphological species *Paracalanus parvus* s.l. was further classified into *Paracalanus* sp. (Slope and Kuroshio) or *P*. *tropicus* (Subtropical) in the metagenetic analysis, according to Hidaka et al. [35].

The species-level comparisons at each station showed that major species in biomass were also detected as MOTUs in the metagenetic analysis with abundant sequence reads (Fig 5). Some non-calanoid species (*Ditrichocorycaeus affinis*, *Sapphirina pyrosomatis*, and *Oithona longispina*) were not successfully compared with MOTUs because of an insufficient reference database for the 28S D2 region. Although the morphological species *Paracalanus parvus* s.l. was detected as one of the dominant taxa at all stations, metagenetic analysis revealed that the dominant MOTU within *P*. *parvus* s.l. was *Paracalanus* sp. at the Slope and Kuroshio stations, and *P*. *tropicus* at the Subtropical stations. As observed at station Slope, species with dominant biomass tended to show high proportions of sequence reads in the metagenetic analysis; however, differences of proportions were also observed between biomass and sequence reads for some species. These discrepancies were mainly observed for large species of copepods with small numbers of individuals, including species in the genera *Eucalanus*, *Haloptilus*, *Pareucalanus*, *Rhincalanus*, and *Subeucalanus*.

## Discussion

### Bioinformatics in MiSeq-based metagenetic analysis

This study confirmed the validity of MiSeq-based metagenetic analysis of copepods, based on comparisons with morphological analysis including non-calanoid copepods and metagenetic analysis using Roche 454. Illumina MiSeq can produce more than 10 times as many sequence reads as Roche 454, and considerably more community samples can be analyzed in a single run using Illumina MiSeq. In addition, paired-end sequences from Illumina MiSeq can provide full-length amplicon sequences with low error rates [15, 16, 36]. The molecular marker 28S D2 region used in this study is approximately 400 bp [21], and high-quality sequences were successfully obtained by 2 × 300 paired-end sequencing using Illumina MiSeq. As indicated by results from bacteria [13, 14] and eukaryotic microbes [18], MiSeq-based metagenetic analysis of copepods would be a cost-effective and high-quality method.

Rare MOTUs with few sequence reads may originate from sequencing error artifacts, and therefore, they should be interpreted with caution [37]. Erroneous MOTUs with few sequence reads are common in MiSeq-based analysis, possibly owing to substitution errors during the sequencing process [15, 16, 38]. In this study, these MOTUs were derived mainly from dominant species, and singleton reads were removed during quality filtering, as suggested by Unno [33]. Without this step, it was difficult to remove all obvious erroneous MOTUs that led to diversity overestimation, because sequence reads of some erroneous MOTUs derived from dominant species were more abundant than those derived from rare species with low biomass. The choice of a high-fidelity DNA polymerase such as the one used in this study is also an important step to reduce erroneous MOTUs, because different types of DNA polymerase affect error rates and chimera formations during PCR [39]. Homopolymer errors are common in Roche 454 [15, 16], and indels were not included for distance calculations to compare data in this study. The sequence length of the 28S D2 region is variable in copepods [21], and insertions and deletions may also increase taxonomic resolution for metagenetic analysis using Illumina MiSeq.

The majority of MOTUs were rare, and abundance thresholds were applied to both MiSeq and Roche 454 analyses. An abundance threshold is common in MiSeq-based analysis and was used in studies of bacterial communities using 16S [13, 38] and fungal communities using Internal Transcribed Spacer 2 [40]. Inclusion of mock community samples helped to determine the abundance threshold [41], and an approximate 0.05% abundance threshold per single sample was used in this study. In this study, we mainly focused on eliminating erroneous MOTUs, and overestimations of diversity derived from possible erroneous MOTUs were reduced by removing singleton reads and rare MOTUs. However, these quality-filtering procedures also risked removing rare taxa in environmental samples. Thus, parameters of quality-filtering steps, including a value for the abundance threshold, should be determined according to the objectives of the study.

### Diversity estimates at a 97% similarity threshold

The 97% similarity threshold is adequate for removing erroneous MOTUs [21, 42], and in this study MOTU numbers were almost comparable between MiSeq and Roche 454 data. MiSeq-based analysis of the mock community showed lower error rates. However, surplus MOTUs were detected in the Roche 454-based analysis even at a 97% similarity threshold (Table 3). Most of the selected species in the mock community were successfully detected in the metagenetic analysis, especially in the Illumina MiSeq data (Table 2). As discussed in a previous study by Hirai et al. [21], *Metridia venusta* (Order Calanoida) was not detected in the metagenetic analyses for unknown reasons, despite successful amplification by Sanger sequencing. Non-selected MOTUs in the mock community were observed in MiSeq and Roche 454 analyses, and were mostly shared between the analyses (S1 Fig), indicating that they were not artifacts (e.g., gut contents of carnivorous species) of the sequencing processes. In field-collected samples, MOTU numbers were lower in the MiSeq- than Roche 454-based analysis (Fig 2; Table 3), which might be related to lower overall error rates in MiSeq-based analysis. Therefore, diversity estimates in MiSeq analysis showed low error rates and outperformed Roche 454 analysis in the reliability of MOTU clustering.

Highly similar taxonomic compositions of copepods were observed between MiSeq and Roche 454 analyses, which concurred with those from the morphological classification (Figs 3 and 4; Table 4). Similar taxonomic compositions in MiSeq and Roche 454 analyses were also observed in bacterial communities [17]; therefore, the use of either of these platforms may not result in significant changes in taxonomic compositions at a 97% similarity threshold. However, we observed platform-specific characteristics of taxonomic compositions in this study. For example, the family Oncaeidae in Poecilostomatoida was detected in MiSeq- but not Roche 454-based analysis of the mock community (Table 2). In field-collected samples, diversity in the Oncaeidae was underestimated, especially in the Roche 454-based analysis, which was also reflected in higher order-level correlations between the morphological analysis and MiSeq analysis than the Roche 454 analysis (Table 4). Thus, the MiSeq-based analysis with respect to taxonomic compositions of non-calanoid copepods was superior. The reference database is incomplete, especially for non-calanoid copepods, as shown in Fig 5, and their phylogenetic relationships are not well resolved compared with those of calanoid copepods [43]. Additional barcoding and phylogenetic studies, especially of non-calanoid copepods, would lead to better taxonomic assignments in metagenetic analysis.

### A strict similarity threshold of 99%

A 97% similarity threshold is sometimes insufficient to discriminate between closely related species; therefore, a strict similarity threshold of 99% has advantages in obtaining high species-level resolution. Both mock and field-collected samples showed high taxonomic resolution in analyses with a 99% similarity threshold, compared with a 97% similarity threshold (Tables 2 and 3). Because nuclear genes might lead to an underestimation of diversity in metazoan metagenetic analyses [11, 44], a higher similarity threshold is recommended for nuclear genes, such as 18S and 28S [10, 21]. In fact, most dominant MOTUs were successfully classified as copepod species in this study, except for some non-calanoid copepods without a reference database (Fig 5). Because the variability of the 28S region differs among taxonomic families of calanoid copepods [43], low variability in a family (e.g., Calanidae and Clausocalanidae) might have led to an increase of MOTU numbers when the similarity threshold changed from 97 to 99% (Table 3). In the mock community analysis, overestimations of diversity were observed at a 99% similarity threshold for MOTU clustering, especially in the Roche 454-based analysis. However, these overestimations were not as prevalent in the MiSeq-based analysis, possibly owing to the high quality of sequence reads in the MiSeq-based analysis. In the field-collected samples, numbers of MOTUs were larger than numbers of morphological species, even in the Illumina MiSeq-based analysis applying a strict abundance threshold in this study (Fig 2). The high sensitivity of the metagenetic analysis can classify immature stages of copepods; in addition, cryptic species of copepods have been commonly observed using the 28S region in the study area [45]. For example, the morphological species *Paracalanus parvus* s.l. was composed of at least three reproductively isolated species in the study area [35], and *Paracalanus* sp. and *P*. *tropicus* were successfully classified among the dominant MOTUs at 99% similarity threshold (Fig 5). In this study, the morphologically unidentified diversity of copepods was detected in the Kuroshio area, where large numbers of copepods, including many rare species, are observed [46], supporting high sensitivity of the metagenetic analysis at 99% similarity threshold. Thus, a 99% similarity threshold provides valuable information with high taxonomic resolution, especially in MiSeq-based metagenetic analysis.

### Compositions of sequence reads in metagenetic analysis

As reported in previous studies of zooplankton communities [8, 21], sequence reads in both MiSeq-based and Roche 454-based methods provide a proxy for biomass. Some discrepancy between biomass and sequence reads might occur from methodological biases including copy numbers of the 28S region and primer mismatches. Morphological data were obtained only from adult individuals in most species, which was not fully compatible with metagenetic analysis including all developmental stages of copepods. Different aliquots of field-collected samples were used for morphological and metagenetic analyses in this study; therefore, the inclusion of large copepods with high biomass might affect discrepancies between biomass and sequence reads, as suggested by species-level comparisons of dominant species (Fig 5). Owing to these methodological biases, the taxonomic composition of sequence reads obtained from metagenetic analysis was inherently not identical to that from morphological analysis. However, results in this study showed that the metagenetic analysis of copepods still provided a valuable quantitative insight to copepod communities with lesser effort than morphological examination.

Quantitative data from Illumina and Roche platforms were highly comparable, especially for abundant sequences [18, 19], and there were also significant correlations between quantitative data from Illumina MiSeq and Roche 454. Concurrently, there were also platform-specific quantitative differences. For example, the proportions of sequence reads were underestimated for Poecilostomatoida and overestimated for Cyclopoida in metagenetic analyses, compared with biomass estimated in morphological analysis. As these discrepancies were especially evident in the Roche 454-based analysis, quantitative analysis of non-calanoid copepods was better represented in the MiSeq-based analysis. However, results in this study are not sufficient to evaluate quantitative data in metagenetic analysis covering various taxonomies of copepods, and further investigation is necessary based on comparison between morphological and metagenetic analyses.

## Conclusions

This study confirmed that previous metagenetic analyses of copepods using Roche 454 data could be successfully replaced by those using Illumina MiSeq data. Although specific bioinformatics steps are required for Illumina MiSeq data, this method was superior to the Roche 454-based method in terms of cost and quality of sequence data. Compared with morphological analysis, including non-calanoid copepods, the MiSeq-based analysis was more similar to expected results than the Roche 454-based analysis in terms of diversity estimates, taxonomic coverage, and quantitative analysis. Lower error rates were observed in the MiSeq- than in the Roche 454-based analysis, especially at a strict similarity threshold of 99%. Thus, MiSeq-based metagenetic methods are a powerful tool for the study of copepod communities in the Kuroshio region. Major taxonomic groups can be comparatively sequenced between Roche 454 and Illumina MiSeq data. However, data from these methods should not be combined and analyzed together, because they produce platform-specific results, especially in the numbers of MOTUs. This study also indicated that bioinformatics procedures, such as abundance and similarity thresholds, should be adjusted according to the specific objectives of each study. To improve metagenetic analysis accuracy, a reference database for copepods is required, especially for poorly represented taxonomic groups such as non-calanoid copepods. A reference database can facilitate accurate species identification of even rare species within zooplankton communities. Sequencing platform and bioinformatics technologies are rapidly evolving, and successive updates of metagenetic methods are expected for future ecological studies of marine planktonic copepods.

## Supporting information

S1 Fig Unrooted neighbor-joining tree for mock community analysis. Reference sequences of 33 morphological species were compared with representative sequences of MOTUs at a 97% similarity threshold in Illumina MiSeq and Roche 454 analyses. Percentages of biomass for species or sequence reads for MOTUs are illustrated in each sequence. Scale bar indicates p-distance.
(TIF)

S1 File Taxonomic file for a naïve Bayesian classifier in MOTHUR. (TAXONOMY)

S2 File Reference sequences for a naïve Bayesian classifier and UCHIME in MOTHUR. (FASTA)

S3 File Reference sequences for add-fragments option in MAFFT. (FASTA)
