# Supplementary figures and images for: Evaluation of metagenetic community analysis of planktonic copepods using Illumina MiSeq: Comparisons with morphological classification and metagenetic analysis using Roche 454
Source: PLoS One. 2017 Jul 17;12(7):e0181452. doi: 10.1371/journal.pone.0181452 (PMC5513544; doi:10.1371/journal.pone.0181452)

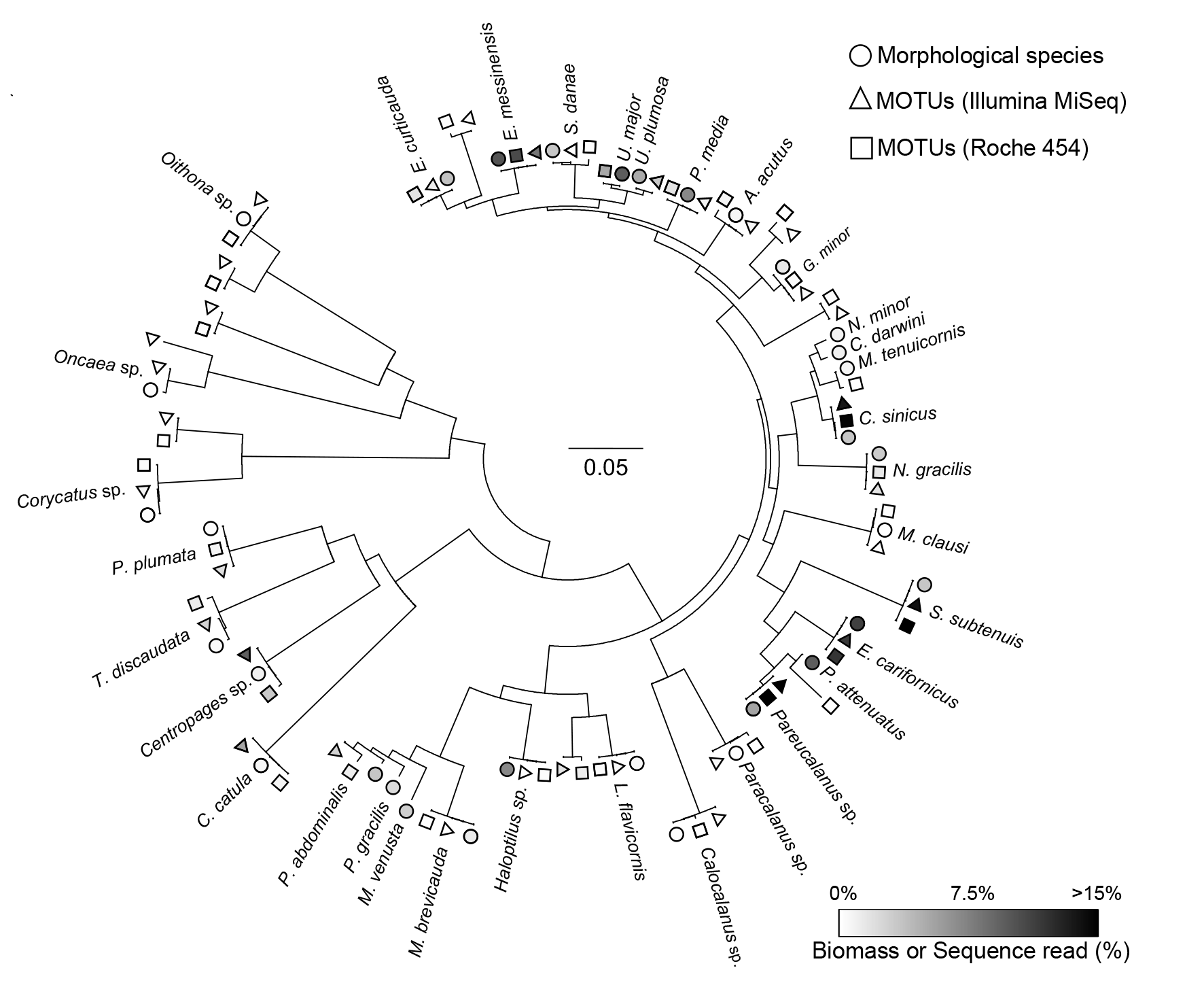

Supplement: S1 Fig — Reference sequences of 33 morphological species were compared with representative sequences of MOTUs at a 97% similarity threshold in Illumina MiSeq and Roche 454 analyses. Percentages of biomass for species or sequence reads for MOTUs are illustrated in each sequence. Scale bar indicates p-distance. (TIF) [file pone.0181452.s001.tif]
